# Missing heritability in Parkinson’s disease: the emerging role of non-coding genetic variation

**DOI:** 10.1007/s00702-020-02184-0

**Published:** 2020-04-04

**Authors:** Jochen Ohnmacht, Patrick May, Lasse Sinkkonen, Rejko Krüger

**Affiliations:** 1 LCSB, University of Luxembourg, Belvaux, Luxembourg; 2 Department of Life Sciences and Medicine (DLSM), University of Luxembourg, Belvaux, Luxembourg; 3 Luxembourg Institute of Health (LIH), Transversal Translational Medicine, Strassen, Luxembourg; 4 Parkinson Research Clinic, Centre Hospitalier de Luxembourg (CHL), Luxembourg, Luxembourg

**Keywords:** Parkinson’s disease, Non-coding variation, Genetic modifier, Gene regulation, Genome-wide association studies, Genetic susceptibility, Polygenic risk scores

## Abstract

Parkinson’s disease (PD) is a neurodegenerative disorder caused by a complex interplay of genetic and environmental factors. For the stratification of PD patients and the development of advanced clinical trials, including causative treatments, a better understanding of the underlying genetic architecture of PD is required. Despite substantial efforts, genome-wide association studies have not been able to explain most of the observed heritability. The majority of PD-associated genetic variants are located in non-coding regions of the genome. A systematic assessment of their functional role is hampered by our incomplete understanding of genotype–phenotype correlations, for example through differential regulation of gene expression. Here, the recent progress and remaining challenges for the elucidation of the role of non-coding genetic variants is reviewed with a focus on PD as a complex disease with multifactorial origins. The function of gene regulatory elements and the impact of non-coding variants on them, and the means to map these elements on a genome-wide level, will be delineated. Moreover, examples of how the integration of functional genomic annotations can serve to identify disease-associated pathways and to prioritize disease- and cell type-specific regulatory variants will be given. Finally, strategies for functional validation and considerations for suitable model systems are outlined. Together this emphasizes the contribution of rare and common genetic variants to the complex pathogenesis of PD and points to remaining challenges for the dissection of genetic complexity that may allow for better stratification, improved diagnostics and more targeted treatments for PD in the future.

## Parkinson’s disease

Parkinson’s disease (PD) is the most common neurodegenerative movement disorder, with a wide range of motor and non-motor symptoms, showing marked differences in age at symptom onset and progressivity (Poewe et al. 2017). The precise etiology of the disease remains largely unknown—both genetic and environmental factors that can lead to PD symptoms have been identified. The accumulation of Alpha-synuclein in Lewy bodies is a major neuropathological hallmark of the disease. Loss of dopaminergic (DA) neurons in the substantia nigra and the associated loss of dopaminergic innervation in the striatum are the main drivers of impaired motor function (Kalia and Lang 2015). The physiological changes resulting in the very varied range of additional non-motor symptoms (such as hyposmia, obstipation and others) are less well understood.

### Phenotypes of Parkinson’s disease

PD patients present with highly variable symptoms. This and the fact that most PD cases are ‘idiopathic’, thus of unknown etiology, poses challenges to clinicians and researchers. Stratification strategies are needed to identify subgroups of patients. Clinically patients can be assigned to different subgroups, using measures such as motor function scores, disease progression rates, cognitive performance indicators, dementia status and the co-occurrence of REM sleep behavior disorder (RBD) (Heinzel et al. 2016). Recent PD cohort studies aim to capture this variability through the integration of detailed clinical information with genetics and additional datasets generated from an extensive range of matched biospecimens (e.g. blood, saliva, stool and post mortem brain samples) (Mollenhauer et al. 2016, 2019; Heinzel et al. 2017; Hipp et al. 2018). This deep phenotyping of PD patients is expected to better inform the selection of individuals suited for specific clinical trials in a true precision medicine approach (Tolosa et al. 2020).

### Genetics of Parkinson’s disease

Only around 5–10% of all PD cases can be attributed to monogenic causes. Mutations in *Parkin* (*PRKN)*, *PTEN Induced Kinase 1* (*PINK1)*, and *DJ-1* (*PARK7*) are linked to early-onset autosomal recessive PD. Mutations in the *Synuclein Alpha* (*SNCA)*, *Leucine Rich Repeat Kinase 2* (*LRRK2)* and *Vacuolar Protein Sorting associated Protein 35* (*VPS35)* genes have been linked to autosomal dominant PD (Klein and Westenberger 2012; Blauwendraat et al. 2020). For another ~ 10% of familial cases, the underlying genetic causes still need to be defined. Sporadic (non-familial) cases make up the remaining ~ 80% of observed cases and the underlying etiology is poorly understood. In sporadic forms of the disease, genetic factors have been identified that contribute significantly to the risk to develop PD. The major risk factors are mutations in *Glucosylceramidase Beta* (*GBA*; e.g. N370S or L444P)) and *LRRK2* (e.g. G2019S), where low penetrance of mutations contribute to the lack of familial aggregation of cases (Nalls et al. 2019).

Many PD-associated mutations display incomplete penetrance or striking variation in expressivity (e.g. age at onset or disease progression rates). This is a common phenomenon observed for most complex traits/disease phenotypes (Cooper et al. 2013). Even for fully penetrant PD mutations, variability in severity and progression of clinical symptoms are common. *SNCA* mutations that are implicated in familial PD can contribute to the risk to develop either PD, PD with dementia (PDD) or dementia with Lewy bodies (DLB) (Guella et al. 2016). Similarly, for carriers of the same *LRRK2* mutation different clinically and neuropathologically defined diseases were described besides PD, e.g. pure nigral degeneration and multiple system atrophy (Zimprich et al. 2004). To what extend the genetic background or genetic architecture shapes disease risk in individuals is still unclear. Also unclear is how environmental factors contribute to the disease and to what extent this depends on an individual’s genetic background.

Interestingly, marked heritability of PD can be observed in some families with no known genetic causes (Blauwendraat et al. 2020). From the observed heritability of PD it is estimated that genetic factors account for up to 30% of identified cases (Nalls et al. 2014). However, this percentage may still increase as our understanding of the genetics of complex diseases improves. The expected presence of yet unknown genetic factors contributing to disease (apart from and in addition to known PD genes) is also supported by the observation of high numbers of phenocopies in familial PD where family members not carrying known PD gene mutations display the same PD symptoms (Klein et al. 2011).

This discrepancy between observed heritability and what can be explained by current genetic knowledge is often referred to as missing heritability. How to approach this missing heritability in the context of complex phenotypes and diseases and how to unravel the underlying genetic contributors has been subject of a wide body of work over the last decades (Manolio et al. 2009; Eichler et al. 2010).

### Known disease-related non-coding variants in Parkinson's Disease

**Fig. 1:**
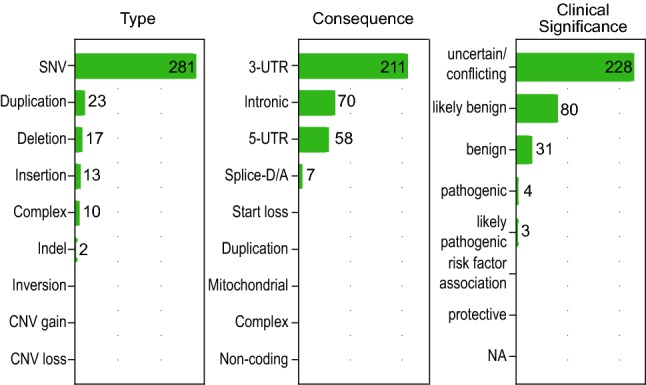
Overview of non-coding variants in ClinVar (27 Nov 2019). Results show the remaining variants after filtering for non-coding regions (‘Splice-D/A’, ‘3-UTR’, ‘5-UTR’, ‘Non-coding’, ‘intronic’), considering only curated variants defined by their reviewer status (‘Criteria provided/multiple submitters, no conflicts’, ‘Criteria provided, single submitter’, ‘Reviewed by expert panel’). Figure adapted from Simple ClinVar results

The genetic factors contributing to PD discussed above are resulting in changes in coding sequences. In contrast, non-coding variation associated with PD is currently understudied. ClinVar (https://www.ncbi.nlm.nih.gov/clinvar/) is the main curated public resource for the assessment of genomic variation in the context of public health and diseases. Using the Simple ClinVar (Pérez-Palma et al. 2019) web interface we investigated the current knowledge about the contribution of non-coding variation in the context of PD. The ClinVar version from Nov 27th 2019 lists for the keyword search ‘Parkinson disease’ in total 1211 variants related to 42 genes. When filtering for variants in non-coding regions and only considering curated variants, 346 variants (28%) related to 21 genes (50%) associated with 21 phenotypes were found (Fig. 1). The only variants annotated as pathogenic or likely pathogenic were six splicing-related variants and only one intronic 164 bp deletion in *PRKN* (Lücking et al. 1998). Most non-coding variants were variants of unknown significance (228 (66%)) and most of them were only found in a single study (329 (95%)). This highlights both the lack of knowledge about the impact of non-coding variants as well as the lack of supporting experimental and genetic data for the characterization of non-coding variants in PD. The chapters below will discuss the potential impact of non-coding variation on gene expression and the establishment of disease phenotypes.

## Complex disease phenotypes and missing heritability

For some phenotypes and diseases, individual mutations with strong effects in single genes can be identified. However, many traits are of a more complex nature involving the interaction of several genes. In these, the associated genetic variants are acting in concert with many additional factors in complex functional networks (Riordan and Nadeau 2017). Genetic contributors to complex phenotypes can be missense variants, splice variants, copy number variants and non-coding variants with regulatory function. The effect of a single variant contributing to a given trait might be very small, as is the case for example in body height, where individual variants influence the phenotype at most by 0.5 cm (Guo et al. 2017). Therefore, the additive effects of several variants are likely required to the shape trait or disease phenotypes.

Work in mice has shown that the observed genotype–phenotype correlation in one defined genetic background is not necessarily transferable to other strains (Sittig et al. 2016). In humans, substantial inter-individual differences in the penetrance of the same mutation defining a disease phenotype can be observed even for monogenic diseases: some mutation carriers may not develop any disease symptoms due to their specific genetic background (Chen et al. 2016). This suggests that in monogenic diseases certain genetic variants or sets of variants can act as modifiers of disease phenotypes. In complex diseases with no single genetic cause, disease modifiers are likely at work, but even more difficult to identify due to the complex interactions shaping the disease-associated phenotypes.

**Fig. 2:**
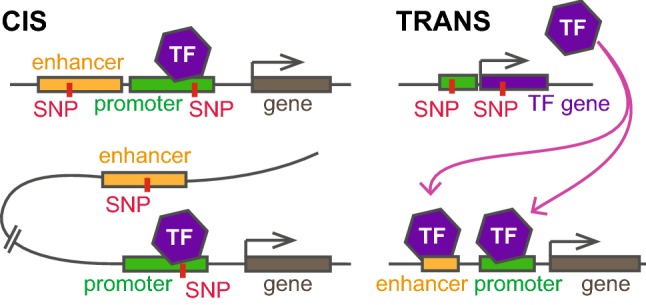
Examples for cis and trans acting genetic variants. Cis acting variants can affect proximal or distal elements resulting in changes in gene expression. Effects in trans alter the abundance or nature of an intermediate factor resulting in altered expression of the target gene. This can be through both coding and non-coding variants

Most of the effects of inter-individual genetic variation are expected to act indirectly, or in trans (see Fig. 2), changing not only coding sequence, or the expression of individual genes, but whole networks of genes and gene products (Cheung and Spielman 2009). Recent estimates from theoretical work suggest that the majority of all phenotypes are shaped by many interactions of low effect size acting in trans (Liu et al. 2019). These effects in trans are much more difficult to identify and to date there is a lack of detailed networks of trans effects.

Genetic variation can play important roles in shaping expressivity and penetrance of certain traits. In complex diseases, many variants are expected to influence several intermediate traits. In the case of monogenic diseases, genetic modifiers are expected to act on the same intermediate traits as the disease-associated mutation. Identifying disease-associated genetic variants and understanding their mode of action is essential to advance our understanding of how complex traits and disease phenotypes depend on the genetic background.

### Strategies to uncover missing heritability

Genome-wide association studies (GWAS) leverage information on recombination events during meiosis in the generation of germline cells. Associating certain recombination blocks to the manifestation of a phenotype (clinical or physiological) allows to identify phenotype/disease-associated common single nucleotide variants (SNVs) or small insertions or deletions (INDEL). GWAS have contributed significantly to our understanding of complex traits and for many diseases, GWAS have uncovered previously unknown disease-associated variants and pointed towards implicated mechanisms (Visscher et al. 2017). However, for complex traits and complex diseases GWAS failed to fully explain heritability. In case of PD, GWAS variants can only explain 16–36% of the observed heritability, based on the to-date most complete meta-analysis covering 17 datasets and over 30,000 PD patients (Nalls et al. 2019). Not included in these heritability estimates are the contributions of rare variants (of any effect size) that can currently not be captured due to the limited sample numbers. Efforts to increase these numbers are underway to expand GWA studies to also include rarer variants with minor allele frequencies of 1% and below. Latest estimates suggest that for this the current sample size will have to be tripled. It is expected that through this effort, additional variants that are currently not reaching genome-wide significance cutoff will make it into the set of identified risk variants (Nalls et al. 2019). In a parallel effort GWAS are for the first time applied to non-Caucasian populations. This will help to overcome the current bias and improve current knowledge on PD genetics through the inclusion of genetically diverse populations, e.g. from India within the Luxembourg-German-Indian Alliance on Neurodegenerative diseases and Therapeutics (Lux-GIANT) (Peterson et al. 2019; Kishore et al. 2019; Schekman and Riley 2019).

As mentioned above, at current sample sizes GWAS fail to capture rare variants. However, increasing evidence suggests that rare variants play a major role in shaping complex traits or complex disease phenotypes (Germer et al. 2019). Whole exome sequencing (WES) is instrumental in the identification of rare disease-associated coding variants that are not captured by GWAS. The Parkinson’s progression marker initiative (PPMI) has generated over 600 whole exome sequences of blood samples of the PPMI cohort including sporadic PD and control subjects (Marek et al. 2011). There are additional efforts ongoing in parallel (Sudlow et al. 2015). WES studies generated mixed results for both sporadic as well as familial PD. While some studies have not uncovered any disease-associated variants, others were able to identify and functionally validate rare disease-associated variants (Farlow et al. 2016; Jansen et al. 2017; Sandor et al. 2017; Shulskaya et al. 2018; Germer et al. 2019). Others point towards risk variants that are shared across PD and Alzheimer’s disease (AD) (Nuytemans et al. 2016). Importantly, none of these studies identified the same rare coding variants. While this is expected for rare variants in the until now limited sample numbers, differences in data processing can contribute to this (Shulskaya et al. 2018)—highlighting a need for standardized analytical pipelines. As will be discussed below, gene expression is highly cell type-specific. Therefore, it is expected that detailed cell type-resolved data from tissues implicated in the disease will dramatically improve future sequencing studies.

Through the dramatic drop in price, whole genome sequencing (WGS) is now applicable to studies with larger sample numbers. In contrast to WES, WGS offers single-nucleotide resolution in both coding as well as non-coding regions. In addition, WGS is untargeted, providing even coverage over the whole genome, alleviating biases introduced by exon-capturing kits (Belkadi et al. 2015). WGS studies leverage information on disease-associated variation together with functional genome annotation (compare Table 1) and predictive scores to narrow down the number of promising candidates for follow up studies. For coding variants, a plethora of in silico tools exist that can be used to estimate the potential deleterious effects of identified variants (Adzhubei et al. 2010; Sim et al. 2012; Kircher et al. 2014). For the biggest part of the non-coding genome no or only limited functional annotation exists. Most often in silico predictions of non-coding variant effects are therefore not feasible or of poor quality. Non-coding variants can, however, be prioritized based on additional functional genomic annotations discussed in the paragraphs below. Due to the small numbers of included participants, WGS studies in neurodegenerative diseases are currently underpowered for the discovery of novel risk variants. Concerted efforts to increase sample numbers are currently underway: The Global Parkinson’s Genetics program (GP2) within the Aligning Science Across Parkinson’s (ASAP) initiative aims to genotype samples from over 150,000 subjects (Schekman and Riley 2019). Importantly, in this effort currently underrepresented populations from e.g. Africa and India are going to be included as well.

**Table 1:** Resources for functional genome annotation, annotations for non-coding variants, and tools for their integration

NIH Roadmap Epigenomics Project	Tissue and cell line resolved epigenome datasets	https://www.roadmapepigenomics.org/
ENCODE	Encyclopedia of DNA Elements	https://www.encodeproject.org/
GTEx	Gene expression/tissue resolved eQTLs	https://www.gtexportal.org/home/
Dropviz.org	scRNA-seq atlas of the mouse brain	https://dropviz.org/
Simple ClinVar	Summary statistic from ClinVar	https://simple-clinvar.broadinstitute.org/
SCAN	SNV and CNV Annotation Database	https://www.scandb.org/newinterface/about.html
AlleleDB	Annotations of cis-regulatory SNVs	https://alleledb.gersteinlab.org/
RegulomeDB	Regulatory element annotations for SNVs	https://regulomedb.org/
Database of Genomic Variants (DGV)	Structural variation in the human genome	https://dgv.tcag.ca/dgv/app/home
FUMA	Functional Mapping and Annotation of GWAS	https://fuma.ctglab.nl/
iPDGC Mendelian randomization portal	Parkinson's Disease Mendelian Randomization Portal	https://pdgenetics.shinyapps.io/MRportal/

### Potential causative variants in GWAS

GWAS studies have identified large numbers of variants associated with complex traits and diseases. However, the identification of underlying causative variants remains difficult. Here, we are summarizing current strategies to identify disease-related variants and genes from common variant studies.

#### Variant fine mapping

One major limitation of GWAS is the use of tag SNVs as proxies for whole recombination blocks and by extension all the co-segregating SNVs (Visscher et al. 2017). It is therefore possible—and more likely than not—that the disease-associated tag SNV is not a causative variant but in fact other SNVs in linkage disequilibrium (LD) are. To improve resolution on the recombination blocks several strategies for fine-mapping of GWAS variants have been developed (Kichaev et al. 2014; Schaid et al. 2018). While fine-mapping strategies help to improve GWAS resolution, it is not alleviating the underlying uncertainty on which of the SNVs identified under a GWAS hit locus might be causative.

One promising approach to combine common and rare variant analyses is to use GWAS results in combination with WES or WGS data. The large amounts of rare variants from WES or WGS can be effectively filtered by prioritizing genetic variants for causality on the basis of preferential linkage disequilibrium (LD). This method assumes that most GWAS signals are not reflecting the actual causal variant, but are only tagging a rare variant in their proximity with a higher effect size variant (Zhu et al. 2012).

#### Quantitative trait loci

One approach to select variants from the overwhelming number of trait-/disease-associated variants is to prioritize them based on information on quantitative trait loci (QTL). QTLs are genomic loci that correlate with a quantitative phenotypic measure such as gene expression (eQTL) or DNA methylation levels (metQTLs). At these loci, certain variants can be associated with altered gene expression or DNA methylation (DNAm) levels. The majority of eQTLs are acting in cis and are predominantly found in promoter elements of correlated genes (Fig. 2). It has been found that disease-associated variants are more likely to be eQTLs (Nicolae et al. 2010). This is in line with the observation that disease-associated variants are more likely to be in gene regulatory elements (GREs; see chapters below). It was shown that eQTLs correlation with gene expression depends on the tissues analyzed (Hernandez et al. 2012). Like gene expression profiles and the associated GREs, eQTLs are highly cell type-specific. This has implications for the identification of functional variants from GWAS. Dependent on the interrogated phenotype, different cell type/tissue-specific eQTL information needs to be considered. Many studies in the past disregarded this need for cell type-resolved functional datasets.

#### Polygenic risk scores

The high number of common disease-associated variants with small effect sizes suggests they could contribute in concert or synergistically to increased disease risk. Polygenic risk scores (PRS) aim to capture this cumulative risk within individuals with the aim to differentiate healthy individuals from patients or individuals at risk (Khera et al. 2018).

While PD age at onset (AAO) GWAS have not uncovered many individual variants with strong effects, work on the cumulative effects of small effect size variants has identified sets of SNVs contributing to the increased overall risk of earlier AAO of PD symptoms (Escott-Price et al. 2015; Pihlstrøm et al. 2016). Interestingly it was shown that the exclusion of all SNVs located in genomic regions with known PD susceptibility loci did not reduce predictive power of the PD polygenic risk scores (Reynolds et al. 2019). Focusing on variants in mitochondrial genes a polygenic risk of small effect variants was shown to contribute to disease risk and AAO in PD (Billingsley et al. 2019). These findings suggest that mainly common variants with small effect sizes (not reaching genome-wide significance in GWAS) are contributing to disease risk. Their concerted action is expected to act on a larger set of pathways and processes resulting in disease. It is however also plausible that rare variants with individual significant impact are shaping this risk. Both scenarios are likely and probably both types of variants contribute together to disease.

PRS currently do provide sufficient predictive power on whether an individual will develop PD. For example, the 1085 variants PRS by Nalls et al. (2019) identifies 14 false positives for one true positive finding, limiting its practical use. Nevertheless, PRS can be useful for patient population stratification: cognitive decline and motor progression over several years could be clearly linked to PRS in PD cohort studies (Latourelle et al. 2017; Paul et al. 2018). A clear polygenic risk has been reported recently to influence differential penetrance observed in PD with *LRRK2* mutations (Iwaki et al. 2020). The most recent approaches include information on rare loss of function variants from WES studies and thereby further improve predictive risk scores (Bobbili et al. 2020).

#### Mendelian randomization

In the context of complex phenotypes/diseases, individual gene expression or protein levels can be considered as intermediate traits contributing to the complex trait. Through mendelian randomization (MR) approaches, causal intermediate traits contributing to the appearance of a complex trait can be identified and distinguished from confounding factors (Neumeyer et al. 2019). Using this information, risk variants can be annotated with genes whose expression they are correlated with. Through this, putative candidates can be selected from the large number of SNVs in LD with the tag SNV. Genetic, metabolomic as well as environmental factors can be considered in MR. In the specific case of GWAS this information can be used to associate LD blocks with their associated regulated genes (Do et al. 2017). Through the integration of tissue-specific DNA methylation and gene expression profiles polygenic risk to PD can be compared to the polygenic risk of another exposure (Nalls et al. 2019; Kolber and Krüger 2019). The recently published MR portal allows to browse two-sample MR results from a wide range of exposures with the latest PD Meta-GWAS analysis (Noyce et al. 2019).

#### Family-based strategies

An alternative strategy to identify disease-associated or modifier variants can be family-based approaches. These methods either use classical linkage analyses, which—similar to GWAS—are based on genotyping data of common variants and then search for haplotype blocks or identity by descent that are following the segregation pattern of a certain trait or disease. Similar to GWAS, this was used in the pre-sequencing era to narrow done regions of interest in large pedigrees. Since the advent of WES and WGS family-based sequencing was also used to identify rare and ultra-rare causal or modifier variants in other diseases (Roach et al. 2010; Schubert et al. 2014; Lalli et al. 2015; Amin et al. 2017). WGS of families allows to detect the segregation of whole shared haplotypes or blocks that are inherited by identity by descent (IBD) for specific traits. These can be used to filter rare disease-causing or modifier variants. Filtering by shared haplotype blocks efficiently narrows down the list of segregating rare and common variants (Roach et al. 2010; Bahlo et al. 2014). Tools like pVaast (Hu et al. 2014) combine linkage analysis and rare-variant association tests from WGS data using family information and different modes of inheritance to prioritize disease-causing variants. Family information combined with GWAS has already been used to detect variants in *DNM3* as genetic modifier for AAO in patients with the *LRRK2*^*G2019S*^ mutation (Trinh et al. 2016).

#### *Gene* × *environment interactions*

The search for unknown genetic contributors to complex traits and diseases is complicated by environmental factors that can affect disease development (Ball et al. 2019). What is more, individual genetic background might shape an individual’s gene × environment interactions (Cavalli and Heard 2019). A thorough review on gene and environment interactions and MR studies in the context of PD can be found elsewhere (Kolber and Krüger 2019).

## Gene regulatory elements

Complicating matters in the search for functional variants is that the vast majority of disease-associated variants identified through GWAS and other methods are located in non-coding regions of the genome. Previously regarded as ‘genetic desert’, it is now understood that the non-coding genome provides important gene regulatory functions (Spielmann and Mundlos 2016). It has been shown that disease-associated non-coding variants are more likely to be located in regions with putative regulatory functions (Maurano et al. 2012), in particular in regions controlling genes expressed and playing important roles in a cell type-specific manner (Hnisz et al. 2013). Understanding the precise effects of non-coding variants on these regulatory regions is essential to understand how they contribute to disease phenotypes by shaping gene expression and downstream pathways.

The principles of regulation of gene expression and the associated changes on DNA and chromatin can be briefly summarized [reviewed for example in (Holoch and Moazed 2015; Allis and Jenuwein 2016; Klemm et al. 2019)]: the expression of a gene is controlled by its promoter and its associated enhancer elements. Promoters serve as the sites for the assembly of the transcription machinery, as a result of the presence of general and sequence-specific transcription factors (TF). TFs can bind to specific motifs of DNA, recruiting other TFs and co-factors to this location. While binding normally occurs in open chromatin, pioneer transcription factors have been shown to be able to also bind motifs in heterochromatin and to recruit additional factors that eventually will result in an opening of chromatin. These pioneer TFs are critical during development. Enhancer elements are short stretches of DNA that can be bound by TFs and as a result, through recruitment of co-activators and interactions with the RNA polymerase complex at the target gene promoter, enhance the transcription of RNA from the target gene. A single enhancer element can contribute to the regulation of more than one gene, and a single gene’s expression can be controlled by several enhancer elements. While this interaction is in cis, the genomic distances between enhancer and promoter elements can be tens to hundreds of thousands of base pairs (compare Fig. 2). These long-range interactions are facilitated by chromosomal looping, which can bring distal enhancer elements in physical contact with their target promoter regions, triggering transcription initiation (Schoenfelder and Fraser 2019). As a consequence, enhancer and promoter elements are integrating spatial and temporal information on TF presence and activation status within a cell into distinct gene expression profiles.

### Identification of gene regulatory elements

**Fig. 3:**
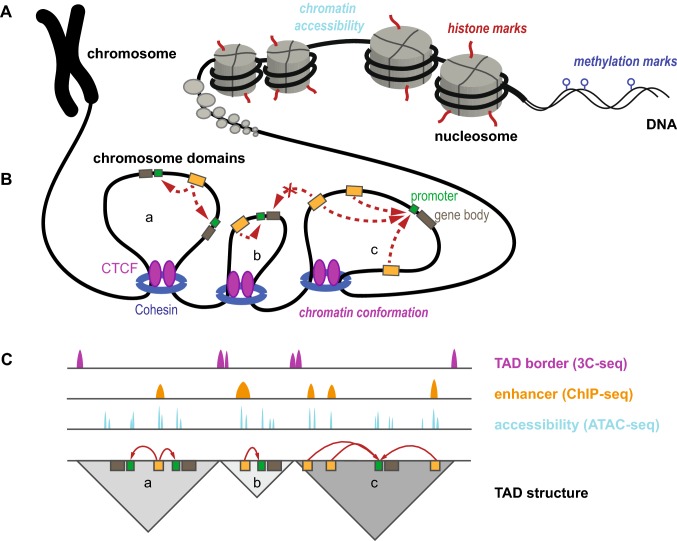
Overview of DNA- and histone-modifications, chromatin conformation and 3D organization of the chromosome. **a** Schematic presentation of DNA- and chromatin marks and chromatin accessibility. **b** Chromatin organization into separated domains, Cohesins and CTCF transcription factors establish domain boundaries. Enhancer and promoter elements can interact within TADs but not with neighboring elements in adjacent TADs. Enhancers are marked yellow, promoters green and gene bodies brown. **c** Idealized next generation sequencing tracks on chromosome conformation capture sequencing using CTCF as bait map TADs, ChIP-seq identifies active enhancer histone marks, and ATAC-seq maps chromatin accessibility. Red arrows indicate how they can be translated into functional annotations mapped to the genome

During cellular differentiation but also in postmitotic cells, covalent DNA- and histone modifications are deposited and removed by writer and eraser enzymes respectively. Reader proteins can further interpret these marks. This is a highly dynamic process essential in the regulation of gene expression (Zhang et al. 2015). Modifications of DNA and histones are often referred to as epigenetic marks. The term epigenetics—originally coined by Conrad Waddington—has received a lot of attention in recent years, but lacks a clear-cut definition (Waddington 1942; Greally 2018). It has been suggested that epigenetics can be separated into two aspects: nature—deposition and removal of DNA and histone marks during regulation of gene expression—and nurture—accumulation of marks as a consequence of environmental exposure, age or disease state (Do et al. 2017). Below, the different features of active gene regulatory elements (GREs) and methods for their identification are outlined (also compare Fig. 3). Typically, a combination of several different approaches is needed for accurate annotation of GREs.

#### Histone modifications

Nucleosomes are built from four different histone subunits that can be covalently modified at specific amino acid residues. Altogether over 100 different modifications of histones are known (Tan et al. 2011). Among the best understood histone marks are methylation and acetylation of specific residues. These are deposited by residue-specific histone-methylases and -acetylases and removed by histone-demethylases and -deacetylases. These marks are highly dynamic and reflect the regulatory state of genomic loci. By analyzing their occupancy, for example, using chromatin immunoprecipitation followed by sequencing (ChIP-seq) with combinations of suitable antibodies, chromatin states such as active or inactive promoters, active enhancers and actively transcribed gene bodies can be identified (Ernst et al. 2011). For example, genomic regions flanked by nucleosomes carrying histone H3 acetylated at lysine 27 (H3K27ac) have been shown to reveal active enhancers engaged in transcriptional regulation (Creyghton et al. 2010; Rada-Iglesias and Wysocka 2011), while poised enhancers that are currently not active lack this mark but harbor histone H3 monomethylated at lysine 4 (H3K4me1).

#### Chromatin accessibility

The accessibility of chromatin can serve as a pointer towards genome regions that are open to interaction with the regulatory machinery (TFs and others) (Klemm et al. 2019). Generally, these regions stretch narrow genomic intervals. Their accessibility can be probed by e.g. DNase-seq (Boyle et al. 2008) and more recently the assay for tagmentation accessible chromatin followed by sequencing (ATAC-seq) (Buenrostro et al. 2013). Within the accessible regions, footprinting strategies allow to identify short intervals that are occupied by TFs (Li et al. 2019b).

#### 3D genome conformation

Chromosomes are organized in a three-dimensional manner inside the nucleus. They are tethered to the nuclear lamina and structured into distinct domains. Using chromosome conformation capture sequencing techniques distal interactions between e.g. promoters and enhancer elements have been identified, including interactions in cis over long distances (Sajan and Hawkins 2012). Knowledge of these interactions are instrumental for the association of putative GREs with their respective target genes. In addition, distinct borders in chromosomes can be identified. It was shown that GREs can interact with each other inside so-called topologically associated domains (TAD), while interaction across TAD borders is rarely observed (Symmons et al. 2014). Borders between the TAD domains are established by Cohesins and the CTCF transcription factor that physically close the domain into a large loop of approximately 1 million base pairs (Fig. 3). The disruption of TAD borders has been reported to introduce pathological changes in cancer and other human disease when GREs can gain aberrant control over additional target genes and, therefore, introduce drastic changes in gene regulation (Spielmann and Mundlos 2016).

#### DNA methylation

High levels of DNA methylation (DNAm) at enhancer elements are usually associated with reduced gene expression from the target promoters (Lea et al. 2018). However, it should be noted that methylation of specific cytosines can also facilitate TF binding, both by repressors as well as activators (Yin et al. 2017). In particular, pioneering factors can bind to methylated DNA and then lead to demethylation of the locus. It is important to note that DNAm is generally a consequence of cell type-specific gene regulatory processes. In addition, environmental factors (for example cigarette smoke) can contribute to differential levels of DNAm. Interestingly, the individual genetic background has been shown to also influence the extent to which environmental exposure or disease conditions can influence the associated differential DNAm profiles (Hannon et al. 2018b). DNAm can be probed using methylation-sensitive microarrays, bisulfite sequencing, or through immunoprecipitation using antibodies specific for methylated DNA (MeDIP) (Weber et al. 2005). More recently, novel technologies such as nanopore sequencing have also enabled the detection of modified DNA bases directly during sequencing (Simpson et al. 2017). Differentially methylated regions are at the heart of epigenome-wide association studies (EWAS) described below.

## Epigenetic changes in neurodegenerative diseases

In addition to their actively controlled role in regulating gene expression, DNA and histone modifications can accumulate over time in response to environmental exposure and disease conditions. This can be referred to as the nurture aspect of epigenetics. Changed metabolism as a result of increasing age, but also of a disease phenotype, could contribute to the differential deposition of these marks. The landmark paper by Horvath has introduced the concept of an epigenetic clock in the form of differential DNAm at specific loci, that can serve as a measure for biological age (Horvath 2013). What has become clear is that certain genomic loci can be subject to changes of these regulatory marks in a disease state, especially so in late-onset diseases such as AD, PD or dementia (Wüllner et al. 2016; Hwang et al. 2017; van Heesbeen and Smidt 2019).

Epigenome-wide association studies (EWAS) aim to identify trait- or disease-associated differential epigenetic marks at specific loci that are a consequence of exposure or disease. Inherently problematic for EWAS is that sample composition needs to be thoroughly controlled to exclude bias through changes in cell populations (e.g. in blood or tissues). Very importantly, since genetic variation has been shown to affect the deposition of DNA and histone marks, disease-associated differential marks need to be controlled for e.g. metQTLs that can be changed as a result of genetics rather than disease or environment (Do et al. 2017). Despite these limitations, EWAS have identified disease-associated changes. DNA methylation marks, for example, are primarily found in enhancer and insulator regions and not in promoter elements, pointing towards function in cell type-specific regulatory processes. This is in contrast to metQTLs that are predominantly found in promoter elements controlling gene expression in a more direct fashion (Do et al. 2017). As discussed before, DNA- and histone modifications are highly cell type-specific. It is therefore crucial that these are not sampled only in the tissues but preferably even in the exact cell types that are contributing to or are affected by a trait or disease. For other diseases and traits DNAm has been investigated in post-mortem brain tissue (Hannon et al. 2018a).

Evidence for concordant PD-associated changes in DNAm in the frontal cortex and peripheral leukocytes has been reported (Masliah et al. 2013), suggesting that blood-derived samples could serve as a surrogate sample for further studies. However, these findings have not been thoroughly validated and were not reproduced so far. In contrast, current studies argue against a high degree of inter-tissue concordance of DNAm marks—with the exemption of age-associated DNAm (Hannon et al. 2015; Farré et al. 2015). Still the use of easily accessible samples may be justified as a potential source for biomarker discovery. Even if these changes are not reflected in brain tissue, DNAm changes in the blood of PD patients could be correlated with symptom progression in several longitudinal studies (Chuang et al. 2019; Henderson-Smith et al. 2019). The promises and pitfalls of epigenetic marks as biomarkers for PD were reviewed in detail recently (Jakubowski and Labrie 2017).

In the case of PD, the obvious candidate tissue for EWAS that would capture any changes in DA neurons is the substantia nigra in the midbrain. However, most studies focused either on easily accessible samples, such as blood or saliva (Kaut et al. 2017; Chuang et al. 2017) or more readily available frontal cortex postmortem tissues (Pieper et al. 2008). PD-related differences in DNAm are also investigated in neuron cultures derived from induced pluripotent stem cell (iPSC) of PD patients and controls. While the methylation marks deposited in iPSC derived cultures are not capturing the nurture aspect of epigenetics, these models are useful in the identification of differential regulation of gene expression as a consequence of genetic background (Fernández-Santiago et al. 2019). A better understanding of PD cell type-specific regulatory networks and the GREs involved in shaping them is needed as a baseline for the identification of PD-specific dysregulation of such networks.

Information on disease-associated changes in DNA- and histone-marks can be leveraged to prioritize distinct regulatory elements for functional characterization. For AD it was shown that certain loci that are differentially methylated between patients and control groups also contain disease-associated variants (Li et al. 2019a). Importantly, also on the level of histone modifications differences have been identified between AD patients and healthy controls (Marzi et al. 2018). In a mouse model of PD, strong association between gene expression changes and differences in histone marks in DA neurons were reported (Södersten et al. 2018). Disruptive variants at these loci could be contributing to disease-associated changes in the deposition of these marks and, in extension, contribute to an intermediate disease phenotype. Such interplay was recently also shown at the *SNCA* locus, where a differentially methylated region in PD patients (Jowaed et al. 2010) was also found to contain PD-associated variants and that this site is a target for DNA demethylases (Sharma et al. 2019). These findings highlight the complex interconnection of regulatory marks and genetics and the inherent difficulty to dissociate environment- or disease-associated contributions from genetic effects on DNA and histone modifications.

## Non-coding variants and their role in gene regulation

Advances in next-generation sequencing technologies and bioinformatic analysis tools are increasingly allowing to test and functionally annotate the non-coding regions of the genome. An extensive body of work has resulted in an increased appreciation of the complex mechanisms by which regulatory elements shape gene expression in a cell type-specific manner. The ENCODE and Roadmap consortia have collected and compared data on gene expression, histone and DNA modifications as well as chromatin accessibility from many different cell types and tissues (Dunham et al. 2012; Kundaje et al. 2015). In their estimates, the majority of the genome serves regulatory functions. One of the important early findings of this work was that accessibility and usage of GREs such as enhancers show high cell type-specificity (Dunham et al. 2012). It was furthermore shown that disease-associated variants are specifically enriched in GREs and even more so in clusters of enhancers, so called super-enhancers, suggesting an important role of gene regulation in disease etiology (Maurano et al. 2012; Whyte et al. 2013; Galhardo et al. 2015). Until now, no computational tools exist that could determine GREs from sequence information alone. Due to their highly dynamic nature and high specificity, cell type-resolved maps of these GREs are required for further investigation of regulatory genetic variation in complex phenotypes and diseases (Hekselman and Yeger-Lotem 2020).

Both common and rare non-coding variants were shown to contribute to differential gene expression in several tissues (Zhao et al. 2016; Li et al. 2017). For frontal cortex, it was shown that approx. 9% of all transcripts’ gene expression levels were regulated by proximal SNPs (Webster et al. 2009) and that 5% of these could be utilized to distinguish between samples from AD patients and healthy controls. More specifically, genetic variants at promoters can result in changed levels of gene expression. This has been shown for the *TERT* promoter in cancer (Fredriksson et al. 2014) and a large body of additional work in other contexts supports this (Deplancke et al. 2016). In addition, it was shown that AD-associated variants in enhancer elements are often found in CTCF binding motifs, suggesting a role in dysregulated chromatin looping and resulting changes in gene expression networks (Kikuchi et al. 2019). Examples of changed gene expression, as a consequence of disease-associated variation altering TF binding at enhancer elements, have been reported for several other diseases and developmental disorders (Karnuta and Scacheri 2018). Importantly, the targets of enhancer variants are not necessarily protein-coding genes but can also be non-coding genes including microRNAs and long non-coding RNAs.

Moreover, SNVs in GREs can change the allele preference, resulting in preferential expression of one allele over the other. This was shown to alter the abundance of coding variant containing transcripts and could serve to explain different penetrance in monogenic diseases (Castel et al. 2018). Differential DNAm at GREs and changes in CTCF binding involved in chromatin structure establishment are discussed as the mechanisms by which variants in non-coding regions can influence allele preference (Wang et al. 2019a).

In summary, non-coding variants can alter gene expression and gene regulatory networks suggesting an important role for non-coding regulatory variants in disease. Below, strategies to identify functional candidates from the vast pool of potential variants will be summarized.

## Leveraging functional genomics to identify disease-associated variants

Translating the vast information of disease-associated genetic variation into a functional understanding of the underlying cellular pathways is challenging. Functional annotation of the genome and increased knowledge on which variants/loci can be associated with certain phenotypic outcomes has advanced this a lot. A list of resources on functional genome annotation can be found in Table 1.

As discussed above, the highly specific use of GREs in different cell types suggests that approaches to identify regulatory variants in complex diseases need to be based on tissue- or cell type-resolved data. Disease-associated variants are enriched in loci with functional histone modifications. Through the integration of cell type-resolved histone mark datasets, it was shown that for certain cell types a higher burden of disease-associated variants can be identified. Disease-associated variants were found to be particularly enriched within GREs of those tissues or cell types that are implicated in the respective diseases, e.g. brain tissue for neuropsychiatric disorders and pancreatic islets for type 2 diabetes (Trynka et al. 2013; Pasquali et al. 2014; Quang et al. 2015). Other work has included information on disease-associated changes of gene expression and chromatin state from animal models. In the case of AD, a clear cell type-specific enrichment of GWAS variants in putative GREs was shown, implicating e.g. microglia as a key cell type in disease etiology (Gjoneska et al. 2015). This work also highlights the value of animal models for the identification of regulatory variants in GREs that are conserved across species.

For PD, a specific enrichment of GWAS variants in active enhancer regions, identified by the presence of H3K27ac, was shown for several different tissues, not only in the brain (Coetzee et al. 2016). So far, no enrichment of PD-associated variants in GREs could be shown for any of the major brain cell types (Nalls et al. 2019; Nott et al. 2019; Reynolds et al. 2019). However, pathway analyses revealed a significant enrichment for variants in certain pathways. These included lysosomal pathways in microglia as well as autophagy pathways in both oligodendrocytes and monoaminergic neurons. GREs associated with mitochondrial gene sets were enriched for PD-associated variants in almost all cell types (Reynolds et al. 2019). While these findings need to be interpreted with caution, as pathway enrichment analysis are difficult to control (Dørum et al. 2009), they nevertheless provide good pointers towards which pathways and cell types to prioritize in future studies. Taken together, non-coding variants were shown to affect GREs active in multiple cell types and are expected to do so in GREs active in less abundant cell types such as DA neurons that have not yet been studied in sufficient depth.

Single-cell RNA-seq (scRNA-seq) allows detailed investigation of gene expression in subpopulations of cells from a disease-associated tissue or cell cultures. Using such an approach AD-associated gene expression changes in individual populations could be identified that were previously disguised by the different expression levels in the bulk sequencing data. While gene expression changes in early disease stages were cell type-specific, in later stages cell types tended to share differentially expressed genes (Mathys et al. 2019). In a recent study, GWAS variants associated with brain disorders and behavioral traits were integrated with cell-type resolved annotations of active enhancers, active promoters and enhancer × promoter interactions. AD GWAS variants were shown to be enriched in microglia-specific regulatory elements, while schizophrenia-associated variants were highly enriched in GREs specific to neurons (Nott et al. 2019). The microglia-specific role of an AD-associated variant containing locus was shown by enhancer deletion. Expression levels of the predicted target gene were reduced, providing evidence that functionally relevant elements can be identified by such a data-driven approach. In the same study, no cell type-specific enrichment of GWAS variants was found for PD. One caveat for these studies is that while bulk midbrain RNA-seq and ChIP-seq datasets exist, few cell type-resolved datasets of PD-affected brain regions like substantia nigra or striatum are available (Welch et al. 2019). Others are from human fetal tissue or mouse tissue (La Manno et al. 2016; Saunders et al. 2018). Most of the work presented here is based on frontal cortex samples. Therefore, potential enrichment of GWAS variants in particular PD implicated cell types (such as DA neurons) or cell type-specific enrichment in certain pathways cannot be excluded yet. Future work on PD-associated variants will depend on better resolved datasets of midbrain tissue, or midbrain specific cellular models.

**Fig. 4:**
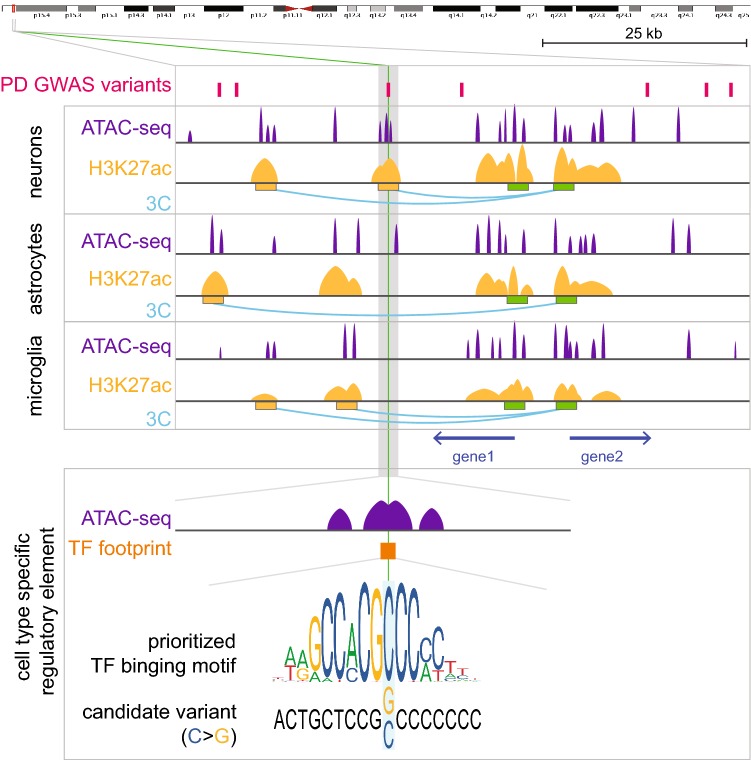
Exemplary prioritization approach for non-coding GWAS variants located in cell type specific gene regulatory element. Cell type resolved information on enhancer marks and chromatin accessibility reduces the number of potential candidate variants. TF footprinting allows to prioritize variants that disrupt TF binding motifs, target genes are identified through chromosome conformation capture techniques. Here an exemplary non-coding variant is identified in an enhancer element that interacts with the promoter of gene 1 in a DA neuron specific manner

Functional annotation on chromatin accessibility can be leveraged to prioritize non-coding variants with putative regulatory roles in known PD genes. Within accessible regions, local drops in next-generation sequencing coverage—footprints—can be identified. These represent TF binding events that can protect open chromatin from digestion or tagmentation during the assay. Within these footprints known TF binding motifs can be found, indirectly revealing the putative TFs that could be binding to these sites (Pique-Regi et al. 2011; Li et al. 2019b). Disease-associated variants disrupting these TF binding motifs are prime candidates for functional testing. Using such an approach, common PD-associated GWAS variants in open chromatin in the *SNCA* locus where prioritized for functional characterization and shown to affect *SNCA* gene expression levels in cultured cells (Soldner et al. 2016). Figure 4 shows an exemplary workflow capturing these prioritization steps.

Chromosome conformation capture (3C) methods have uncovered many enhancer × promoter interactions. Using this information, putative GREs containing candidate variants from GWAS, EWAS or other sources can be linked to specific promoter regions and the likely regulated target genes. A striking example of how chromosome conformation annotation can aid the identification of functional non-coding variants comes from a GWAS of elevated body mass index. One of the strongest associations was found at the *FTO* gene locus. However, candidate variants were found to disrupt a repressive TF binding site within a GRE that controls expression levels of *IRX3* and *IRX5* located over one million base pairs away (Claussnitzer et al. 2015). Using a similar approach, a hypomethylated locus at the *IGF2* gene, identified in neurons of patients with schizophrenia or bipolar disorder, was shown to physically interact with the promoter of the tyrosine hydroxylase (*TH*) gene, coding for the rate-limiting enzyme in dopamine synthesis. Hypomethylation of DNA at this enhancer element was associated with increased *TH* expression levels, providing an explanation for the high levels of dopamine and the observed benefits of DA receptor antagonist treatment in psychosis (Pai et al. 2019). In the context of neurodegenerative disease GWAS, cell type-resolved 3C data was used to identify cell type-specific target genes for individual GREs that contained AD-associated variants (Nott et al. 2019). While their datasets contain deep information on PD, the authors did not elaborate on the effects of PD-specific variants.

Enhancer × promoter interaction maps are not available for many cell types. Computational approaches associating accessibility of GREs based on functional genomics data and gene expression are currently being developed to provide GRE to target gene predictions. Importantly, these tools do not rely on prior assumptions as to which genes might be regulated by a GRE. Also, sets of GREs controlling the expression of a particular gene can be identified. Using such approaches, enhancer × target gene networks can be built, from which non-coding variants from GWAS involved in the regulation of disease-associated genes were identified (Shooshtari et al. 2017; Cao et al. 2017).

The work presented above is mainly focused on the identification of individual candidate variants, contributing to the expression of individual target genes. However, higher-level effects on gene regulatory networks might also contribute to the development of disease. As previous work showed, non-coding variants are accumulating in hubs of gene regulation. Understanding how deregulation of these hubs and their associated gene regulatory networks can contribute to disease phenotypes requires prior knowledge on the healthy state of these networks. Using information on gene expression and enhancer elements these networks can be computed (Saint-André et al. 2016; Gérard et al. 2019). It is conceivable that non-coding variants can disturb these networks, and, to some extent, be responsible for the aberrant gene expression observed in the disease state (Gao et al. 2018). Functional validation of sets of non-coding variants would require combinatorial activation or repression; the means to do so are currently being developed and discussed below.

## Systems biology approaches

Systems biology approaches aim to integrate datasets from different sources on a network level. Especially in case of complex diseases such as PD, with the very broad range of symptoms and implicated tissues, a systems-level approach can contribute to a more generalized view of the pathological changes. The concepts and necessary tools are reviewed elsewhere (Parikshak et al. 2015; Glaab 2018). For example, using gene set enrichment analysis and topological pathway models, disease-associated changes on cellular pathways can be identified. For PD this was done based on transcriptome data of substantia nigra tissue, in which significant PD-associated changes in several pathways were found (Zheng et al. 2010). Network-based pathway enrichment analysis on human transcriptome datasets was used to identify shared pathways between PD and ageing (Glaab and Schneider 2015). Genes previously known to play a role in DA neuron development and PD (e.g. *NR4A2*) were found to be differentially expressed in both ageing and PD. A likely shared role of underlying gene regulatory processes was proposed. Expanding on this work, gene expression network modules were used to define cell type-specific signaling modules active at neuronal synapses. In these *STMN2* was identified as the top regulated gene and subsequently shown to be important for endocytosis in mouse DA neurons. A knockout of *STMN2* resulted in reduced numbers of DA neurons in mice (Wang et al. 2019b).

Due to the complexity of the datasets, the resulting networks and models can be difficult to interpret. Machine learning approaches recognize patterns in data that can serve to classify distinct groups. Recent work has introduced a genome essentiality score through the integration of a wide range of datasets covering 3D genome organization and enhancer annotation. This score was then shown to serve as a strong indicator of the relevance of individual deletions in enhancer elements (Wells et al. 2019).

Functional genomics datasets have helped to understand complex diseases and point at players at critical network locations. The integration with clinical phenotypes and additional deep phenotyping data on a systems level will, however, be needed to fully understand how disease phenotypes develop and how they can potentially be detected even before clinical manifestation.

## Validation of disease-associated variants

Due to the large numbers of non-coding variants that can be associated with diseases and the laborious process involved in functional testing, candidates need to be prioritized. The integration of functional genomics data allows to exclude many variants that are not located in putative GREs. Cell type-resolved data can provide further insight in which cell types variants likely act in. However, without experimental validation in vivo or in vitro, these predictions are of limited use. Variants with predicted effects only in a certain cell type would require testing in appropriate cell culture or animal models. For coding variants, functional readouts are rather straightforward: Transcript and protein abundance as well as protein function can be measured. This is not the case for non-coding variants, where, owing to the variability of potential roles, no single application exists with which effects could be assessed.

### Model systems

The work reviewed above highlights the highly cell type-specific engagement of GREs. In addition, the genetic background in which non-coding variants are acting is expected to influence its effect. Therefore, the functional characterization of potential disease-associated variants should be performed in genetically defined experimental systems that ideally recapitulate the genetic background in which they were identified. The advent of patient-derived induced pluripotent stem cells (iPSC) has made this possible (Meissner et al. 2007; Takahashi et al. 2007) and protocols to differentiate e.g. DA neurons from iPSCs have been described (Kriks et al. 2011; Reinhardt et al. 2013). During reprogramming of somatic patient cells to iPSCs, most DNA and histone modifications are erased and reset to a pluripotent status. This is an essential aspect of successful reprogramming (Grzybek et al. 2017). During differentiation from iPSC to certain lineages in vitro, the majority of the GRE that are part of the nature aspect of epigenetics will be re-established. Work in mice has shown that midbrain-specific DA neurons derived from iPSC show a methylation profile similar to primary DA neurons isolated from midbrain tissue of the same mouse line. This indicates that iPSC-derived neurons can serve as a suitable model to investigate most of the physiologically relevant GREs in vitro (Roessler et al. 2014)*.* DNA methylation profiles for DA neuron cultures derived from human iPSC lines have been generated (Fernández-Santiago et al. 2015, 2019). While specific DNAm patterns unique to PD where identified in DA neuron cultures, there is to date no study that compared methylation profiles in matching sample pairs of human neurons derived from both iPSCs and the brain. This again highlights the need for well-planned cohort studies where clinical data, genome sequence and functional annotations as well as biospecimens should be available for deep phenotyping and disease modeling.

Animal models are also used to validate the functional consequences of deletion or insertion of candidate GREs. In mice, an *IGF2* locus enhancer element interacting with the *TH* locus was shown through deletion to be required for *TH* expression (Pai et al. 2019). Other work used an enhancer element reporter strategy in mouse and zebrafish to identify DA neuron-specific candidate GREs at the *SCNA* locus, containing common PD-associated non-coding variants (McClymont et al. 2018).

### Tools to test non-coding variants

Classical approaches to assess genomic regions ability to control gene expression are reporter assays with minimal promoter elements driving expression of a reporter gene (Barakat et al. 2018). These allow the interrogation of many different elements and a proxy quantification of gene regulatory function. In addition to promoter elements, also individual enhancers harbor transcriptional initiation sites and can be cloned adjacent to specific reporter sequences, allowing to test their functionality in any cell type of interest. Although such assays lack the native chromatin context and long-distance looping interactions, they have been successfully applied for massive parallel high-throughput screening of millions of putative enhancer sequences in different human cell types (van Arensbergen et al. 2017). The same methodology has also been applied for in vivo testing of human enhancer sequences carrying known SNVs and has allowed validation of the impact of tens of thousands of SNVs on gene expression (van Arensbergen et al. 2019).

While reporter assays allow high-throughput analysis, experiments in the native chromatin context are needed for detailed understanding and validation of individual variants. In recent years, clustered regularly interspaced short palindromic repeats (CRISPR)/Cas9 systems have been widely employed for targeted gene editing. Through guide RNAs (gRNAs), the Cas9 protein can be targeted with high specificity to a distinct locus on the genome (Adli 2018). Gene editing using CRISPR/Cas9 technology now serves as a gold standard to investigate the effects of specific non-coding variants (Soldner et al. 2016; Guo et al. 2017). Importantly, genome editing allows to investigate the effects of individual variants in a defined genetic background.

Novel developments include catalytically inactive or “dead” versions of Cas9. These are no longer able to introduce DNA strand breaks but can still be targeted to specific loci using gRNAs. CRISPR activator (CRISPRa) or inhibitor (CRISPRi) versions of dCas9 have been generated by fusion with repressor or activator domains (such as KRAB or VP64 respectively) allowing to assess the function of putative GREs in selected loci (Pulecio et al. 2017). Both classical Cas9 knockin/knockout as well as CRISPRa and CRISPRi are already used in targeted applications and in screening approaches where large genomic regions are interrogated for putative enhancer elements (Fulco et al. 2016; Gasperini et al. 2020). A database for CRISPR/Cas9 screens has been established (Rauscher et al. 2017). Ideally, in the future genome-wide annotation of all GREs and their targets in a cell type-resolved manner will be available for future systems-level analysis. Work in parallel uses saturation mutagenesis in disease-associated regulatory elements to identify positions where single nucleotide variation has strong impacts on function (Kircher et al. 2019). Their results suggested that current tools that predict non-coding variant impact have poor predictive value.

With a suitable functional readout (such as cell survival, growth rates or gene expression) gRNA libraries targeting all known transcription start sites (TSS) can be screened on a genome-wide level. These screens are superior to siRNA screens with respect to knockdown efficiencies and already provide insights into the effects of individual gene expression on certain phenotypes. A CRISPR knockout screen in a *PARKIN*-GFP reporter cell line identified novel transcriptional regulators (repressors) of *PARKIN* (Potting et al. 2017). As it was not the scope of this particular study, no information on the regulatory elements involved was provided. Similar approaches could be applied to other disease-associated mechanisms, pathways or phenotypes in vitro (Callif et al. 2017). While CRISPR/Cas9 methods do not provide single-nucleotide resolution, they hold the promise of combinatorial testing of regulatory elements—allowing to probe sets of disease-associated candidate regions in concert.

## Outlook

Recent years have seen increasing interest in the non-coding genome and its contribution to the regulation of gene expression. It is becoming clear that genetic variation in these parts of the genome can contribute to disease risk and variability of observed disease phenotypes. With the emerging availability of high throughput genotyping data first light will be shed into the yet missing heritability of complex diseases like PD. This will allow to translate the complex genetic architecture of this common neurodegenerative disorder into stratified treatment strategies. The integration of multi-omic datasets with clinical data will allow to better understand the complex interplay of genetic variation, genetic background, and environmental factors resulting in disease-associated deregulation of gene expression, disease pathways and phenotype.

In an initial step, increased sample sizes of GWAS and next-generation sequencing datasets will provide sufficient resolution to common and rare (non-coding) variants contributing to diseases. Already now, single cell-resolved datasets on gene expression and functional genomics make it possible to identify cell type-specific molecular pathways with a higher burden of disease-associated variants and to point at particular cell types with increased overall variant burden. In the case of PD, the inclusion of datasets covering DA neurons and other cell types, from affected tissues like the substantia nigra, will answer whether there are specific cell types in PD with increased risk variant burden contribution to disease etiology. Recent studies already hint at the important role of non-coding variants in shaping individual gene expression and disease risk. The identification of cell type-resolved gene regulatory networks and how they are perturbed in the disease state will be a crucial next step to achieve. Through the development of computational models that can integrate this information, the effects of sets of non-coding risk variants on such a network could be identified. This would provide additional information for the genetic stratification of patients, to better select individuals that can benefit from certain treatment options and to identify candidates for precision medicine clinical trials.

To tackle the complexity of the disease, future work in PD will also have to integrate additional layers, such as microbiome, metabolome and exposome at a systems level.
